# Perceptions and Expectations of Youth Regarding the Respect for Their Rights in the Hospital

**DOI:** 10.3390/children11020222

**Published:** 2024-02-09

**Authors:** Roberta De Rosa, Maria Anna Siano, Angelo Colucci, Anna Giulia Elena De Anseris, Paolo Siani, Pietro Vajro, Giulia Savarese, Claudia Mandato

**Affiliations:** 1 Department of Medicine, Surgery and Dentistry “Scuola Medica Salernitana”, Pediatrics Section, University of Salerno, 84081 Baronissi, Italy; roberta.derosa@unimi.it (R.D.R.); m.siano14@studenti.unisa.it (M.A.S.); acolucci@unisa.it (A.C.); pietro.vajro@gmail.com (P.V.); 2 University Hospital San Giovanni di Dio e Ruggi d’Aragona, 84131 Salerno, Italy; annagiulia.deanseris@sangiovannieruggi.it; 3 Pediatrics, AORN Santobono-Pausilipon Children’s Hospital, 80122 Naples, Italy; p.siani@santobonopausilipon.it; 4 Department of Medicine, Surgery and Dentistry “Scuola Medica Salernitana”, Psychology Section, University of Salerno, 84081 Salerno, Italy; gsavarese@unisa.it

**Keywords:** adolescents, children, drawings, healthcare, hospitalization, humanization of care, interview, narration, patient-centered care, self-determination

## Abstract

Information obtained from children themselves regarding the characteristics of the ideal hospital that ensure well-being during a hospital stay is scarce. Here, we report the opinions, perceptions, and expectations of 700 children and adolescents about their experiences, assessed through a mixed-method research approach with age-appropriate questionnaires, three open-ended questions, and an analysis of optional pictorial and textual narratives. Most children indicated that, while they acknowledged the expertise of hospital staff, they also noted several shortcomings, e.g., insufficiently understandable medical information as well as emotional and cognitive support. The continuity of schooling and the right to suffer as little as possible were also critical issues. Adolescents valued in particular the quality of care and services provided, the hospital’s adherence to equality and non-discrimination rights, and protection systems but negatively perceived several aspects related to play and participation. Significant differences in the co-occurrences of the most frequently used text terms with the keywords “hospital” and “child/adolescent” between age groups highlight variations in the way patients perceive and articulate their experiences within the hospital setting depending on the cognitive processes linked to age. In drawings, prevailing attention was placed on the physical context of the hospital room, with figures expressing mostly negative emotions. Specifically, in this regard, the main emotion in children was sadness, and, in adolescents, it was fear. Overall, these insights are pivotal in the context of our research objectives as they shed light on the nuanced preferences, needs, and perspectives of children and adolescents during their hospital stays. Recognizing the identified shortcomings, we propose recommendations emphasizing the improvement of medical communication clarity, enhancement of emotional and cognitive support, and the improvement of programs to avoid instructional gaps during hospital stays. Addressing these specific needs is critical for a more comprehensive approach to pediatric healthcare provision.

## 1. Introduction

“Humanization of care” (HOC) is a still-indistinct concept, lacking well-defined dimensions, related to a new way of conceiving medicine. In extreme synthesis, it refers to an approach in healthcare that prioritizes the holistic well-being of patients by considering their physical, emotional, psychological, and social needs. It involves treating patients with dignity, respect, empathy, and compassion and actively including them in their own care. Humanizing care recognizes that healthcare is not just about treating diseases or symptoms but also about addressing the individuality and autonomy of each patient [1,2]. In pediatrics, HOC is an even more articulated concept since it implies the provision of targeted assistance not only to the patients but also to their entire family, who are the recipients of care [1,3,4,5]. Hospitalization can serve, therefore, as a useful testing ground for HOC, given that it is an unpleasant experience which represents a challenging trauma across all ages. In pediatric age, the impact may be particularly negative especially because children may not yet be fully equipped with the appropriate cognitive tools to rationally process the negative emotions felt during an unpleasant experience. 

Numerous studies, healthcare charters, committees, and policy documents in line with the United Nations Convention on the Rights of the Child [6] have emphasized that the aforementioned concepts add to the significant stressor of a possible separation from their family. Insufficient information about hospital procedures and decision-making processes [7] as well as medical professionals’ use of difficult-to-understand technical language [8] have been reported by the scarce prior research, which has been based mainly on interviews and questionnaires examining how children and adolescents perceive and expect to have their rights respected in hospitals. Aloneness, fear, rage, sadness, missing home, and anxiety are the most commonly reported emotions [9]. Hospitalization always results in a state of discontent [10] that causes stress for the child and their family as well [11]. While play and recreational activities could help relieve some negative feelings and provide a sense of normalcy and control in children, they are not always sufficient or available [12,13]. Recognizing the areas that require improvement to assure the well-being of pediatric patients during hospitalization is, therefore, a pivotal aspect of HOC. Notably, existing information obtained by adults (parents/visitors or staff members) as proxies for children [14,15] needs to be viewed with caution as children’s and parents’ experience of hospital stays do not always match [16]. In an Italian project in which various humanization interventions were implemented in the pediatric wards of the seven regional hospitals located in the Campania region, we showed that the level of existing humanization issues perceived by users (parents/visitors) was frequently different also from that perceived by staff members [17]. However, in our previous study, we did not explore the point of view of young hospitalized patients and/or directly listen to their opinions. 

Therefore, the present study aimed to complement our previous findings with the objective of providing a comprehensive snapshot of the respect for children and adolescents’ rights assessed directly from their voices when admitted to pediatric wards. Specifically, we aimed to assess opinions, perceptions, and expectations about their experiences through a mixed-method research approach with age-appropriate questionnaires, open-ended questions, and textual/figurative narratives describing their state of mind and the ideal hospital, as they would like it to be. These features were explored also through drawings, a recognized developmentally appropriate method which may enable pediatric patients to communicate their experiences even better than with words [18,19,20].

## 2. Materials and Methods

This study was conducted on 700 children and adolescents (350 children aged 6–11 y.o. and 350 adolescents aged 12–18 y.o.) hospitalized in the general pediatric wards of seven hospitals in the Campania region, each contributing with 50 respondents. Compliance with their rights in hospitals was assessed using two questionnaire tools specifically intended for children and adolescents. Both tools were derived from the “Manual and Tools for the Assessment and Improvement of Children’s Rights in Hospitals” [21,22,23] prepared by the Task Force on Health Promotion for Children and Adolescents, a working group of the International Network of Health Promoting Hospitals and Health Services, in collaboration with hospitals and international partners, including the World Health Organization [24]. Briefly, both tools were based on seven standards ± substandards inspired by the 1989 Convention of the Right of the Child [25], charters, and working documents. The assessment tool used for younger children aged 6–11 years (“Children’s Rights in Hospital and Health Services: Assessment Tool”) consisted of 16 questions, each with three answer options: “yes”, “no”, and “do not know”/“not applicable”. The assessment tool used for older patients (“Children and Adolescents Aged 12–18”) consisted of 61 multiple-choice questions with three similar answer options, organized in 6 standards and 15 substandards (see Supplementary Files S1 and S2) [21,22,23,26]. For each standard, the child or adolescent could also express their opinion as a short comment. The questionnaire tools for both age groups included three additional open-ended questions regarding the (1) positive aspects of the hospital, (2) negative aspects of the hospital, and (3) improvement proposals for the hospital. 

The percentage of affirmative answers for each substandard was calculated [21,22,23,26]. According to the authors of the questionnaires [27], the questionnaire research trainee did not have a “typical” profile but was a medical student or pediatric fellow who was familiar with the issues and had experience in facilitating such process. For adolescents in particular, the research trainee was instructed to be a “neutral” person so that the young person could calmly respond without judgment and with sincerity. If they wanted, the teenagers could answer in writing. During interviews, the statements were read slowly to the children, and the children responded via written and/or verbal communication (as in Foster [15]).

In addition to the two specific questionnaires mentioned above, children and adolescents were invited to a direct interview with an open question and open answer consisting of a textual (verbal answer) or a figurative (drawing) narrative. Both were optional (not mandatory) and were considered here to support the data obtained from the questionnaires. The open question was twofold: (1) “describe or draw your state of mind during these days spent in the hospital”, and (2) “describe or draw your ideal hospital as you would like”. For figurative narration, the patients were requested to convey their thoughts through drawing and were given the freedom to choose whether or not to verbally comment on their pictures, both during the execution and afterwards. The interviewer refrained from providing specific instructions in this regard.

Individual interviews were planned prevalently in the morning, at varying times for each patient, to minimize interference with ward activities, in the place preferred by the children or adolescents (hospital room, playroom, etc.). The patients and their parents received a thorough explanation from the research trainee regarding how the process would take place and how the results would be used. The patients were consistently accompanied by their respective parents/caregivers, each of whom provided written informed consent for their participation in this study. Continuous presence of the parent/caregiver throughout the entirety of the interview process was not deemed imperative. 

The thematic content of verbal answers provided by the children and adolescents was analyzed using the T-Lab Plus textual analysis software (T-lab plus 2020.1 version 5.1.1.3), a natural language processing technique which allows one to evaluate the weight of a term (lexical unit) within a document (context unit) [20]. Briefly, through automatic algorithms, the software reduces words to their base or lexical root (i.e., the lemma), selects the lexical units with the highest occurrence values, and selects the words with the highest values of term frequency/inverse term frequency (TF-IDF) or applies the chi-square test to all the crosses of each selected word. For the present study, the textual corpora of the interview reports were submitted for a thematic analysis of elementary contexts. Key-word occurrences and co-occurrences were examined in relation to the strengths of textual analysis software. In the resultant radial diagram and pictures elaborated by the software, the size or distance from the center of words that had been lemmatized three times is proportional to the frequency of their co-occurrence.

The analysis of drawings was commented using criteria previously described in the literature [28,29,30,31,32]. Each individual drawing describing either the State of mind during hospital stay or the Hospital I would like [ideal hospital] was examined looking at the atmosphere in the scene as well the emotions and relational quality of the drawing features. Drawings were then labeled with the following:

- the gross description of the content, along with details about colors, drawing size, and relationships between people;
- the assessment of basic and social emotions (i.e., fear, sadness, anger, embarrassment, shame, shyness) and the emotions felt when they moved to the level of representation and imagined their ideal hospital (i.e., joy, surprise/security). Finally, drawings with similar categorized emotions were grouped together.

The positive and negative emotions conveyed through the drawings were correlated with the outcomes of the questionnaire concerning the perception, attention, and management of pain by healthcare professionals (responses to questions 14, 15, and 16 administered to children aged 6 to 11 years). Due to the non-normal distribution of the data and sample sizes, the association between these variables and the statistical significance were evaluated using non-parametric statistical methods, i.e., the Spearman’s Rank correlation coefficient and the Wilcoxon-Mann–Whitney U test, respectively [28].

## 3. Results

Table 1 summarizes the patients’ diagnoses and hospital stay durations prior to interview. The mean age and male-to-female sex ratios were 8.0 ± 1.7 years and 55.7%/44.3% in the first group and 13 ± 1.3 years and 51.4%/48.6% in the second group. 

In most interviews, the parents or guardians stayed with the children and/or adolescents; they were occasionally absent during this period in only a few cases. As the participants did not object to any of these situations, the interviews proceeded smoothly and lasted for 15 min and 25 min on average in children aged 6–11 years and those aged 12–18 years, respectively. Data were collected at diverse times of the day, most frequently in the morning and sometimes in the afternoon. Only a minority of children participated to the open question and open answer interview [textual narrative (*n* = 84) or figurative narrative (*n* = 144)]. The adolescents mostly refused to participate in the proposed figurative narrative activities.

### 3.1. Age-Specific Assessment Tools

#### 3.1.1. Tool Questionnaire for Children Aged 6–11 Years

The right to be accompanied by one’s parents at all times and during all medical procedures had the highest percentage of affirmative responses (Table 2). Another guaranteed right to receive almost all affirmative answers was the right to play; in most cases, the participants were with other children or adults and were rarely alone (Table S1). Important critical issues included the children’s inability to understand the words of doctors when they were speaking directly to them about their disease (70%). With respect to the right to information, more than 70% of the children reported that their mothers explained the reasons for their hospitalization. Continuity of schooling for hospitalized children was guaranteed in less than 50% of cases, with critical percentages in one of the structures examined, in which there was no school in the hospital.

#### 3.1.2. Tool Questionnaire for Children Aged 12–18 Years 

The right to be accompanied by parents, equality and nondiscrimination, and pain management were the substandards that achieved the highest percentage of affirmative answers (“yes”) in all seven hospitals (Table 3). Conversely, privacy, game, information, and participation were the substandards that obtained the highest percentage of negative answers (“no”) in all seven departments analyzed (Tables S2–S7).

### 3.2. Open Questions 

Verbal answers to open-ended questions were obtained by 84 participants. Some commonalities existed between the responses of the two age groups: both appreciated the hospital staff’s expertise, had a negative opinion on sanitary facilities, and agreed on the need to improve environmental comfort, entertainment and leisure, and hygiene (Table 4A,B).

### 3.3. Drawings Analysis

Drawings were obtained from 144 children; Table S8 summarizes which were the positive (N = 80) and negative (N = 64) feelings. Overall, the analysis of drawings highlighted an important difference between the emotions felt by the patients when they remained on their level of perception because they were experiencing hospitalization (i.e., fear, sadness, anger, embarrassment, shame, shyness) and the emotions felt when they moved to the level of representation and imagined their ideal hospital (i.e., joy, surprise/security). The patients’ prevailing basic and social emotions during hospitalization fluctuated, ranging from sadness/pain/fear to general well-being (Table 5). 

**Figure 1:**
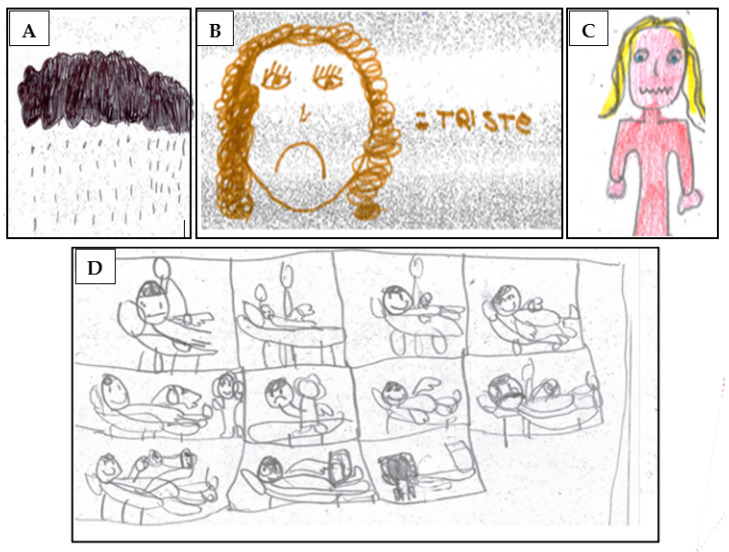
(**A**) Black cloud; (**B**) sad (triste) face; (**C**) zigzag mouth/face; and (**D**) information and participation in routine procedures.

A few example drawings describing sadness/fear as the prevailing emotion during hospitalization are shown in Figure 1A–C, with the black cloud with rain denoting a deeply negative experience of sadness and/or pain (Figure 1A) and with the mouth drawn as an inverted U (Figure 1B) or at a zigzag (Figure 1C) representing the mood swings experienced during hospitalization.

The child in Figure 1D recognized the health worker and actions taken by other professionals who came to check his vital signs and administer the therapy. The child went through a series of fluctuating emotions: he was initially intimidated by the doctor’s approach and subsequently calmed down. However, immediately afterwards, he was frightened again as soon as the intravenous drug infusion was performed; thereafter, he was found to smile again (Figure 1D).

**Figure 2:**
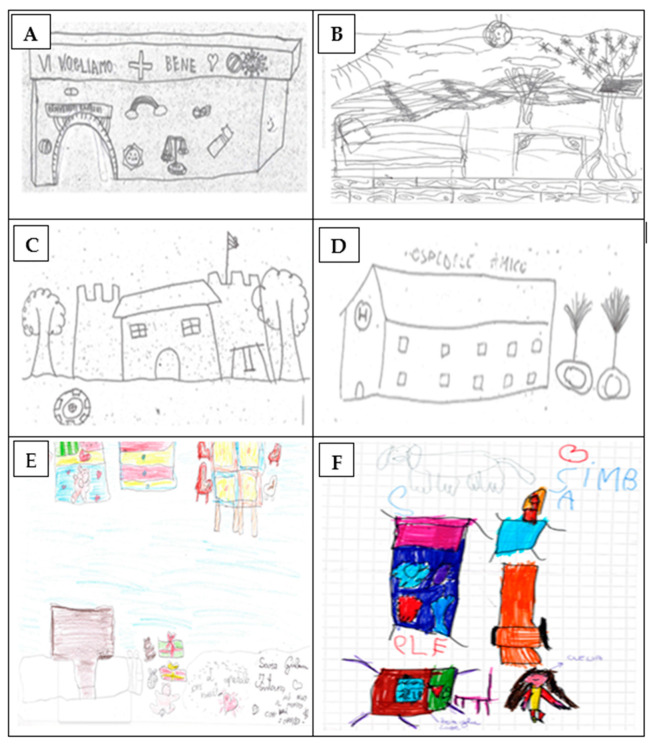
(**A**–**F**) Selected drawings regarding the ideal hospital. The individual subfigures are explained in the text.

In some drawings pertaining to the ideal hospital, the prevailing emotion was joy and trust inherent in all playful elements represented (Figure 2A–F). The main emotion was welcome, which was evident from the sentence “we love you”/“Vi vogliamo bene” (Figure 2A) or the absence of a building as the hospital structure (Figure 2B). The hospital was represented in the open to outline a certain rejection of the hospital environment. The hospital structure, which was created between two towers, appeared as a solid castle, thereby conveying a sense of stability and security (Figure 2C). Trust was evidently the predominant emotion in writing (hospital friend/ospedale amico) (Figure 2D). Interestingly, the majority of children presented a double bed (Figure 2E). In some drawings, bright colors stood out, and their contents seemed to represent the objects to which the little girl in Figure 2F was emotionally linked, such as a small dog.

Table 6 presents the main commonalities found in the children’s drawings.

A statistically significant association was found between positively expressed emotions through the drawings and a greater perception of attention to pain received by the healthcare professionals (question 14: R^2^ 0.62 *p* = 0.046; question 15: R^2^ 0.71 *p* = 0.039; question 16: R^2^ 0.67 *p* = 0.043).

### 3.4. Narrative Content Analysis

**Figure 3:**
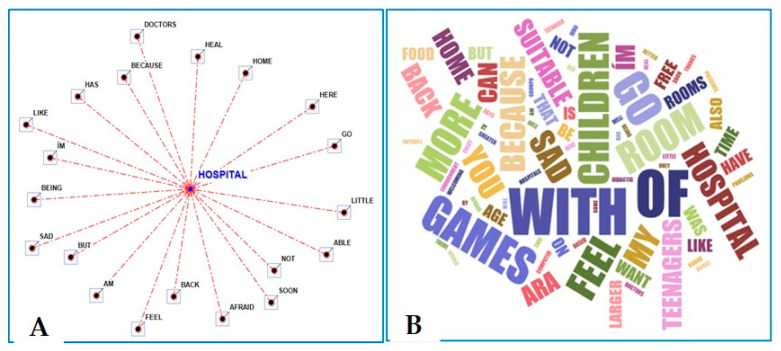
Radial diagram with the selected lemma (hospital) placed in the center (**A**) and word cloud picture (**B**) when describing the hospital and your mind (ages 6–11 and 12–18) (words’ closeness to the center and their sizes are proportional to the frequency of citations) [33].

As part of the description of one’s own mind, an analysis of textual narration data allowed for a further evaluation of how the children and adolescents perceived their hospitalization. The terms most frequently used together with the selected key term “hospital” were as follows: heal, home, little, back, not, sad, soon, feel, afraid, and doctors (Figure 3A), as well as sad, free, back, feel, games, home, with, room, children, teenagers, and suitable (Figure 3B).

**Figure 4:**
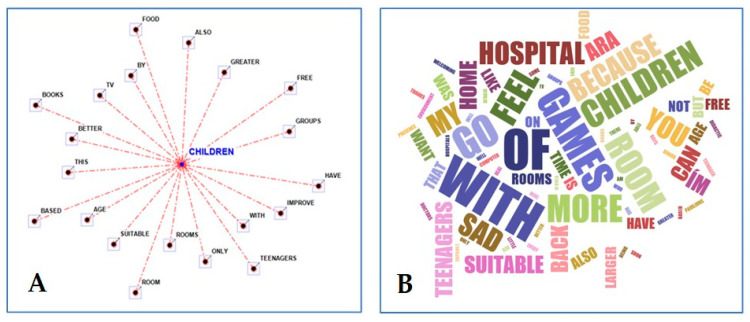
Radial diagram with the selected lemma (children) placed in the center (**A**) and word cloud picture (**B**) when describing the ideal hospital (words’ closeness to the center and their sizes are proportional to the frequency of citations) [33].

As for the description of their own ideal hospital, the terms most frequently used together with the keyword “children” by the group aged 6–11 years were as follows: free; groups, improve; rooms; age, books; TV; and food + suitable. (Figure 4A,B).

**Figure 5:**
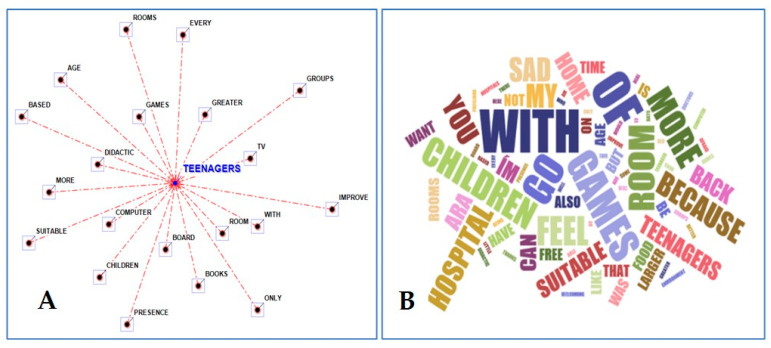
Radial diagram with the selected lemma (teenager) placed in the center (**A**) and word cloud picture (**B**) when describing the ideal hospital (words’ closeness to the center and their sizes are proportional to the frequency of citations) [33].

The words that were predominantly used together with the key term “teenager” by the group aged 12–18 years were as follows: games; children; teenagers; with; rooms; free, go; you; feel; welcoming; group; better; time; home; and with (Figure 5A,B).

## 4. Discussion

The tools used in the present study could provide useful information for assessing children’s perspectives during their hospitalization, an area that has not yet been entirely and adequately explored in a holistic manner. The responses to questionnaires, drawings, and narration analyses, collectively, allowed us to explore at the same time different perceptions and expectations of youth regarding their rights during hospitalization. Through interviews with hospitalized children and adolescents, there were twenty narrative clusters relating to the evaluation of their stay and what their ideal hospital is, even if only with imagination. It emerged that, despite adequate respect for their rights in the majority of the examined pediatric wards, there is still a large space to intervene to achieve a real complete HOC. This appears to stretch from multiple perspectives, requiring sensitivity and attitudes which go beyond the pure specialization or professional skills of healthcare professionals.

The specific data gained from the children’ questionnaires and open-ended questions point towards the existence of a number of deficiencies that require improvements while acknowledging the hospital staff’s expertise in most cases. In particular, these pertain to the need to provide patients and sometimes their families with shared and adequately understandable medical information as well as emotional and cognitive support for any doubts or uncertainties. Assurance of the continuation of school education and the right to suffer as little as possible appear also as critical issues. Although guaranteeing young patients the possibility of staying with their families at every stage of hospitalization and the possibility of playing was respected in all cases, this situation needs to be improved. As indicated by the CRC document [25], all of these issues are of extreme relevance as they can represent an effective response to guarantee that the traditional clinical approach of exclusively pharmacological diagnosis and therapy is overcome and that greater attention is paid to the promotion of psycho-pedagogical initiatives.

Commenting on the questionnaires responses of adolescents is more difficult because of the multiple substandards in each standard. Overall, the substandards with the highest percentages of affirmative responses in this study’s wards pertained particularly to the quality of care and services offered, compliance with the rights of equality and non-discrimination, and the existence and implementation of a child protection system in the hospital. Conversely, the substandards with the highest percentage of negative ratings common to all the departments analyzed regarded privacy, games, information, and participation, which represent three important aspects of an adolescent’s life. The latter aspects, in particular, confirm the literature data according to which suboptimal children’s involvement in the care process and participation in decision making lead to dissatisfaction [7,10,34] because they are either not receiving sufficient information about procedures [35] or have to struggle with incomprehensible terminology used by health professionals [8]. Globally, as revealed by the data analysis, adolescents have significantly different health needs from children, and health services should better protect their privacy and personal autonomy.

The direct interview with a verbal description of the children’s mood during hospitalization and their ideal hospital confirmed that hospitalization had a great impact on the children’s social interactions, as they were estranged from their relatives, friends, and school. “I want to go home, I feel sad,” “I miss my sister,” “I’m sad, I’m afraid of not being able to heal,” “I am feeling lost,” “Sad,” and “Sad! I wish there was a more familiar environment (hospitals are sad)” were among the most emblematic phrases. By performing an analytical study of word co-occurrence networks, we explored, measured, and mapped various types of relationships between key terms, which further helped us to reveal the perception of hospitalization and one’s ideal hospital. From the drawings, it emerged that the term “hospital” was most frequently associated with others related to healing, the desire to return home soon, sadness, fear, and doctors. Most of these feelings during hospitalization mirror those reported in other studies that found stress [11]; solitude, fear, anger, and sadness [9]; and worry, missing home, and anxiety [36] to be related to being in an unfamiliar environment [37,38,39].

The word co-occurrence analysis approach focusing on pediatric hospitalization has been used only in a few studies that are not easily comparable. Some studies have used a projective technique (e.g., the revised Barton Hospital Picture Test (BHPT)) [40] where researchers present a non-specific stimulus (picture) and ask the patient to tell a story about it. Responses to pictures are thought to provide insights into children’s inner feelings, perceptions, and fears [41]. Similar to our findings, however, in general, children’s stories with this projective approach reflect fears of being alone in the hospital, fears of known experiences, and feelings of being threatened by uncertain possibilities [9]. Children tend to respond to these concerns by asking to have their parents next to them, to take familiar items to the hospital, or to go home [7,38,42]. Nurses are expected to provide more care and support [42,43,44,45]. In some BHTP stories, hospitalized children said that they were bored when they were alone; they needed adults or other children for play, conversations, or simple company. Games, play, and playrooms have often been cited positively regarding hospital stays. As a result, hospitalization is not always traumatic if they can make new friends and have more play equipment [36,44,46,47,48]. In contrast to our study, none of the previous studies conducted word co-occurrence analyses. The co-word analysis method produces a preferable research situation in terms of matrix dimensions and clustering results [49]. By capturing the semantic meaning of words and their co-occurrence, compared to others, our study allowed for the exploration, measurement, and mapping of words relationships that were useful to gain insight into how ideas or topics are connected in children’s/adolescents’ language. In particular, the textual analysis helped us to more clearly identify and support earlier research indicating that a stressful mix of loneliness and homelessness, fear, rage, discontent, melancholy, and anxiety affects both children and adolescents during their hospital stays. When considering what kids and teens would have liked to see in their ideal hospital, it is interesting to note that, despite some differences, some similarities also surfaced, including an improved environment which would have allowed them to stay in groups and have more freedom.

Drawing a description of their moods during the days spent in the hospital and of their ideal hospital was essential for telling the full story. With children’s limited vocabulary and cognitive capacity, through which they have to express their thoughts, emotions, and feelings, acquiring an effective means of communication is essential [50]. Drawing has often been regarded as the universal language for toddlers [51] and has been shown by some studies to help patients in sharing their emotions of anxiety, pain, fear of death, and desire for normality [50,52,53]. Figurative narration is an acknowledged tool for exploring the impact of hospital stay on children’s minds, enabling patients to express their emotions and moods and tell stories better through images than with words. Some details considered apparently marginal while looking at the questionnaires or verbal narration turned out to be important when represented with images. For instance, although the parents of younger patients always remained close to their children during the day and night, they often had to share the bed with the child or sleep on chairs or armchairs, thus highlighting the critical issue that they were not allowed to rest adequately. For children, this situation can represent a source of further discomfort and sadness, as evidenced by the pictorial representations which describe their ideal hospital, in which there is almost always a room with a double bed which can accommodate both the child and the parent (Figure 2E). Remarkably, this provides an opportunity to ponder the pros and cons when refurbishing or designing brand new pediatric wards with almost exclusively private rooms. In fact, while this may be attractive to parents and adolescent patients, it may hinder the socialization needs of school-age children, an issue strongly felt by children during their hospital stay [54].

The analysis of the emotionally expressive aspects of children’s drawings is a very open issue because drawings may be analyzed from more than one aspect. In fact, they can be the manifestation of specific personality traits interpreted within the theoretical framework of psychoanalytic theory and its derivatives. However, they also represent an attempt to devise and scientifically validate a classification of emotional indicators [28], and, last but not least, they represent a communicative tool that describes personally important or emotionally significant topics that exceed the capabilities of abstract linguistic expressions. The latter was the aspect that we examined—that is, considering drawing as the result of the interaction of sensibility/emotions, perception, and motor functions with the factor of social experience [55]. Separating the emotions a child drew during their hospital stay from those that were a result of their own typical personality can be challenging. Some factors to think about during this process include the following: a. the drawing’s context, revealing how the child is reacting to being admitted to the hospital, particularly if it explicitly shows the hospital setting, medical procedures, or elements of the child’s hospital stay, and b. the emotional impact of being hospitalized rather than being a more accurate representation of the child’s personality or past experiences, if the drawing expresses feelings of fear, anxiety, or sadness in relation to the hospital setting. It emerged that both fundamental emotions (e.g., anger, fear, sadness), which are the most frequent emotion, and social emotions (e.g., embarrassment, shame, shyness) were prevalent. As far as the ideal hospital results are concerned, overall, they are in line with those obtained by others who, using semi-structured interviews mediated by drawing and analyzed by inductive thematic analysis, found, in the designed hospital, combined elements and material resources from the physical environment and elements of comfort and well-being [18,56]. Overall, our results indicate that drawings can guide interventions aimed at improving children’s hospital stay, reducing pain, and reducing stress and anxiety.

The present study has some limitations that require further investigations describing hospital experiences. Similar to other studies which aim to give children a voice, our study inevitably suffers from being somewhat mixed in tone and content to accurately reflect the children’s views [57]. The children who participated in this study were mostly Caucasian/White, Italian, reflecting the community in which the study was conducted. Therefore, the issues described may lack generalizability and not reflect the views on hospitalization of either more- or less-privileged, ethnically diverse children. Children with developmental delays and those admitted to the emergency department or to certain specific subspecialty units, such as diabetes, oncology, or trauma, were also not represented [9]. We, therefore, caution that the results could likely be quite different for these other patient groupings. Also, the more or less continuous presence of parents/caregivers and the interview setting/variable degree of participant confidentiality and privacy measures might have influenced the responses and might, therefore, represent a potential limitation to be taken into due account. Other limitations regard the unavailability of specific frequencies of each medical diagnosis and details on the severity or burden of treatment. However, we considered our patient sample to be likely homogeneous because of the usual same need of intensity of medical care pertaining to general (not specialized) pediatric wards. We also acknowledge that obtaining verbal answers only from some participants who were willing to collaborate left out other respondents, possibly affecting the accuracy of our data, e.g., leading to undercoverage bias. Additionally, we could not ask follow-up questions about the specific co-occurrences/relationships between the themes that emerged. It is certainly possible that participants could have had their own interpretations about why certain themes were related. Nevertheless, these relationships between the themes emerged regardless of prompting. Lastly, although we took steps to make our research more reproducible and replicable with detailed explanation of how participants were recruited and the prevalent use of descriptive rather than statistical analyses, we acknowledge that convenience sampling can introduce several types of unresolved research bias. The strength of this study lies in its comprehensive approach to exploring the pediatric hospitalization experience. The use of diverse methods, including drawings, to accompany the traditional grounded theory methods of questionnaires and narrative analyses allowed for a more detailed understanding of children’s perspectives, emotions, and needs. This mixed-methods approach not only generated quantitative data but also provided qualitative insights directly from the children. By incorporating the voices of both children and adolescents, this study offers a well-rounded view, considering the unique needs of each age group. This holistic methodology contributes to a thorough and insightful examination of the pediatric healthcare environment, providing valuable information for enhancing practices and settings.

## 5. Conclusions and Study Implications

Overall, our study acts as a way to think about the humanity of the hospital experience for children and adolescents but should not be seen as taking us to conclusions from which we can draw practice or policy direction. Nonetheless, the results outlined a broad-spectrum of information on patients’ views regarding the main barriers to HOC in a group of pediatric wards in the Campania region, which may be useful for planning future improvement interventions. These should include the need for a careful assessment of what exists and how it is perceived by the children themselves, attempting to recreate the environment that they desire, effectively contributing to the promotion of well-being, and dispelling the myth that the hospital environment is cold and hostile. Overall, the results of our study are consistent with those obtained by others in most of the prevalently thematic investigations that we summarized in the scoping review.

It appears that children and adolescents wish to decide how to be treated and how to take care of themselves, and special attention should, thus, be given to conveying information through a language that is understandable and appropriate for the age, development, and maturity of the minor. As recommended by others, the information must concern both the contents of diagnostic or therapeutic decisions and the consequences of their non-execution in case of refusal of consent or withdrawal of previously provided consent [58,59]. Within the interdisciplinary healthcare team, it would probably be necessary to integrate child psychologists and behavioral health specialists into the healthcare team, implementing targeted programs to enhance the mental well-being of children and adolescents, for example, those able to integrate play components into routine hospital activities [60]. Future studies should fully examine strategies for humanized care implementation and quantitatively verify their effectiveness, rethinking the cultural approach of hospital environments and the layout of healthcare settings so that they are truly patient-oriented or, even better, “child-friendly.”

Overall, there appears to be a need (1) to create a holistic therapeutic model that considers the introduction of transversal skills that all operators should acquire and (2) build a hospital environment that supports the overall well-being of children, covering the mental, physical, and emotional needs of children and adolescents, including due attention to play, continuity of the school curriculum, and painless procedures. In this regard, some children reported that the pain felt is not only a physical one but also an emotional one, induced above all by the distance from their loved ones who, in this scenario, represent the fixed point of young patients. All of these aspects should be taken into due account also while considering that the needs of children are different from those of adolescents and adults. Regarding teenagers, an important point reported by all is the need to create separate dedicated spaces for children that safeguard privacy and independence while allowing them to meet their peers or read and watch movies together.

Taking into account the number of limitations of our study, future research should aim for more diversity, especially among populations with developmental delays or specific medical conditions, to enhance generalizability. One could explore strategies to encourage broader participation and ensure more representative insights to avoid potential undercoverage bias in verbal responses from willing participants and consider more standardized frameworks for drawing analysis.

## Figures and Tables

**Table 1:** Diagnoses and hospital stay duration of the 700 children.

	6–11 Years *(n* = 350)	12–18 Years (*n* = 350)
Main clinical diagnoses	acute diarrhea, asthma, febrile seizure, salmonella gastroenteritis, bronchitis, pneumonia, urinary tract infection, otitis media, viral illness, purpura	acute and chronic diarrhea, pneumonia, bronchitis, otitis media, trauma, urinary tract infection, eating disorder, febrile viral illness, infectious mononucleosis.
Hospital stay duration before interview	5 ± 3 days	6 ± 1 days

**Table 2:** Tool questionnaire 6–11 years (N = 350).

Question	% Yes µ (± SD)	% No µ (± SD)	% N.A. µ (± SD)
1. Can you please let us know how your stay in hospital was for you?	100 (±0)	0 (±0)	0 (±0)
2. Did you play while you were in hospital?	85.8 (±8.2)	14.2 (±8.2)	0 (±0)
3. Who did you play with? ***			
4. Did you have the opportunity of going to school in the hospital?	25.7 (±21.1)	73.6 (±21.8)	0.7 (±1.3)
5. Do you like the school in the hospital?	20.7 (±17.3)	3.9 (±5.8)	75.4 (±19.1)
6. Did anyone tell you why you came to hospital?	84.4 (±15.3)	15.6 (±15.3)	0 (±0)
7. Did the doctor explain why you were hurting/what was wrong with you?	65.7 (±25.3)	33.3 (±25.5)	1 (±1.5)
8. Did you understand what s/he said?	63.3 (±15.2)	22.3 (±10.5)	14.4 (±13.5)
9. Did someone tell you how you can get better?	79.6 (±13.4)	16.3 (±10.9)	4.1 (±8.8)
10. Do you feel comfortable saying if something is making you unhappy in the hospital?	61.7 (±21.3)	36 (±21.4)	2.3 (±4.1)
11. Do you know who to talk to if you are not happy in the hospital?	86.9 (±16.3)	12.9 (±15.6)	0.2 (±0.8)
12. Were your parents always with you during your stay in hospital?	99 (±1.3)	1 (±1.3)	0 (±0)
13. Did your parents stay in the hospital overnight?	100 (±0)	0 (±0)	0 (±0)
14. Have you felt pain while you were in the hospital?	65.1 (±24)	34.9 (±24)	0 (±0)
15. Did anyone ask you if you were feeling pain?	84.4 (±13.4)	15.3 (±12.3)	0.3 (±0.8)
16. Did anyone try to make the pain better?	79.4 (±17.1)	13.6 (±13.7)	7 (±5.5)
Total Rights	73.6 (±24.1)	19.6 (±19,1)	6.7 (±2.4)

*** see Table S1; N.A., not available.

**Table 3:** Questionnaire 12–18 years. Answers obtained for each standard * (N = 350).

STANDARD	% Yes µ (± SD)	% No µ (± SD)	% N.A. µ (± SD)
1: Quality services for children	63.40 (±8.05)	28.54 (±5.35)	8.06 (±5.19)
2: Equality and non-discrimination	55.71 (±7.92)	17.61 (±8.32)	26.68 (±6.69)
3: Play and learning	44.04 (±12.66)	39.55 (±9.79)	16.41 (±7.35)
4: Information and participation	57.36 (±8.91)	32.55 (±9.70)	10.09 (±5.66)
5: Safety and environment	76.00 (±8.68)	11.96 (±5.64)	12.04 (±5.66)
6: Pain management & palliative care	76.79 (±9.89)	16.43 (±6.85)	6.79 (±4.05)

* Answers to the specific substandards pertaining to each of the six standards are shown in Supplementary Tables S2–S7. N.A., not available.

**Table 4:** (**A**) Answers to three open-ended questions (children) N = 32. (**B**) Answers to three open-ended questions (teenagers) N = 52.

(**A**)					
**POSITIVE THINGS**	**% µ (±SD)**	**NEGATIVE THINGS**	**% µ (±SD)**	**IMPROVE/** **IDEAL**	**% µ (±SD)**
Hospital staff expertise	65.14 (±13.10)	Sanitary facilities	29 (±17.8)	Environment comfort	36 (±10.84)
Playroom	31.5 (±2.12)	Food	45 (±10.8)	Entertainment/leisure	43.16 (±20.30)
I do not know	23.16 (±13.69)	I do not know	48.33 (±14.43)	Hygiene	19.5 (±11.44)
				Food	21.66 (±20.82)
(**B**)					
**POSITIVE THINGS**	**% µ (±SD)**	**NEGATIVE** **THINGS**	**% µ (±SD)**	**IMPROVE/** **IDEAL**	**% µ (±SD)**
Hospital staff expertise	49.86 (±10.2)	Sanitary facilities	19.83 (±8.25)	Environmental comfort	23.33 (±5.77)
Reception	22.5 (±3.53)	Food	28.2 (±13.64)	Entertainment/leisure	36.6 (±23.09)
Environmental comfort	6.3 (±3.05)			Hygiene	12.5 (±5)
Patient care	4.5 (±0.70)				
Kindness/courtesy	15.8 (±11.90)				
Quiet environment	5 (±0)				
Playroom	20 (±13.23)				

(**A**) Children were asked to give their opinion on which were the good/bad things during their hospital stay and what they felt was necessary to improve for it to be close to their ideal. (**B**) Teenagers were asked to give their opinion on which were the good/bad things during their hospital stay and what they felt was necessary to improve for it to be close to their ideal.

**Table 5:** Basic and social emotions in children’s drawings.

“State of Mind during Hospital Stay”		“Hospital I Would Like” [Ideal Hospital]	
BASIC EMOTIONS	SOCIAL EMOTIONS	BASIC EMOTIONS	SOCIAL EMOTIONS
Fear (Figure 1B,C)	Embarrassment	Joy (Figure 2A–F)	Stability/Security (Figure 2C)
Sadness (Figure 1A,B)	Shame	Surprise	Welcome (Figure 2A)
Anger	Shyness		Trust (Figure 2D)
Fluctuating (Figure 1D)			

Numbers and letters in parentheses refer to examples depicted in Figure 1 and Figure 2.

**Table 6:** Qualitative synopsis of the commonalities in the children’s drawings.

**Colors**	- Warm (Yellow, Orange, Pink, Red) - Cold (Black, Gray, Brown).
**Drawing size**	- It develops in height - It occupies only the lateral spaces of the sheet - It develops only in the lower and upper part of the sheet - Presence of curved lines - The drawing takes up all the space on the sheet
**Size relationships between people**	- Short and non-existent legs compared to the body - Triangular head and of different dimensions

## Data Availability

The data presented in this study are available upon reasonable request from the corresponding author. The data are not publicly available due to specific ethical and privacy considerations.

## Supplementary Materials

The following supporting information can be downloaded at https://www.mdpi.com/article/10.3390/children11020222/s1: Supplementary File S1 Children’s rights in Hospital and Health Services: Assessment Tool for Children aged 6–11—Edited by: Ana Isabel F. Guerreiro March 2012; Supplementary File S2: Children’s rights in Hospital and Health Services: Assessment Tool for Children and Adolescents aged 12–18—Edited by: Ana Isabel F. Guerreiro March 2012; Table S1: Question 3: Who did you play with?; Table S2: Questionnaire 12–18 Years Standard 1: Quality Services For Children; Table S3: Questionnaire 12–18 Years Standard 2: Equality And Non-Discrimination; Table S4: Questionnaire 12–18 Years Standard 3: Play And Learning; Table S5: Questionnaire 12–18 Years Standard 4: Information And Participation; Table S6: Questionnaire 12–18 Years Standard 5: Safety And Environment; Table S7: Questionnaire 12–18 Years Standard 6: Pain Management And Palliative Care; and Table S8: Types of Feeling in the 144 children’s drawings.
