# Supplementary material for: Perceptions and Expectations of Youth Regarding the Respect for Their Rights in the Hospital
Source: Children (Basel). 2024 Feb 9;11(2):222. doi: 10.3390/children11020222 (PMC10887615; doi:10.3390/children11020222)
Supplement: Supplementary file 1 [file children-11-00222-s001.zip › SUPPLEMENTARY FILE S1 TOOL 6-11.pdf]

children

MDPI

SUPPLEMENTARY FILE 1 Children's rights in Hospital and Health Services:
Assessment Tool for Children aged 6-11 - Edited by: Ana Isabel F. Guerreiro March 2012

1. Can you please let us know how your stay in hospital was for you?
Comments
Yes
No
Not applicable/ don't know

2. Did you play while you were in hospital?
Comments
Yes
No
Not applicable/ don't know

3. Who did you play with?
Comments
Yes
No
Not applicable/ don't know

4. Did you have the opportunity of going to school in the hospital?
Comments
Yes
No
Not applicable/ don't know

5. Do you like the school in the hospital?
Comments
Yes
No
Not applicable/ don't know

6. Did anyone tell you why you came to hospital?
Comments
Yes
No
Not applicable/ don't know

7. Did the doctor explain why you were hurting / what was wrong with you?
Comments
Yes
No
Not applicable/ don't know

8. Did you understand what s/he said?
Comments
Yes
No
Not applicable/ don't know

9. Did someone tell you how you can get better?
Comments

Yes
No
Not applicable/ don’t know

10. Do you feel comfortable saying if something is making you unhappy in the hospital?
Comments
Yes
No
Not applicable/ don’t know

11. Do you know who to talk to if you are not happy in the hospital?
Comments
Yes
No
Not applicable/ don’t know

12. Were your parents always with you during your stay in hospital?
Comments
Yes
No
Not applicable/ don’t know

13. Did your parents stay in the hospital overnight? Where?
Comments
Yes
No
Not applicable/ don’t know

14. Have you felt pain while you were in the hospital?
Comments
Yes
No
Not applicable/ don’t know
15. Did anyone ask you if you were feeling pain?
Comments
Yes
No
Not applicable/ don’t know

16. Did anyone try to make the pain better?
Comments
Yes
No
Not applicable/ don’t know

Last question
What have been the **good things** about the services provided here?

What have been the **bad things** about the services provided being here?

If you were in charge, what are the **first things you would change** to make things better for children and adolescents?

THANK YOU
Printed name and initials of Interviewer Post held Signature of Interviewer Date
