# Supplementary material for: Perceptions and Expectations of Youth Regarding the Respect for Their Rights in the Hospital
Source: Children (Basel). 2024 Feb 9;11(2):222. doi: 10.3390/children11020222 (PMC10887615; doi:10.3390/children11020222)
Supplement: Supplementary file 1 [file children-11-00222-s001.zip › SUPPLEMENTARY FILE S2 TOOL 12-18.pdf]

children

MDPI

SUPPLEMENTARY FILE S2

Children's rights in Hospital and Health Services:

Assessment Tool for Children and Adolescents aged 12-18 Edited by: Ana Isabel F. Guerreiro

March 2012

Standard 1: Quality services for children

All services provided to children aim at delivering the best quality possible care, by taking into account clinical evidence available, the respect of children's rights and patient and family's views and wishes.

Sub Standards

1.1. The hospital/health service ensures that all institutional activities are based on the best evidence available and that staff are adequately trained.

1.1.1. Do you think that you received the best care here?

Comments

Yes

No

Not applicable/ don't know

1.1.2. Do you feel you are included in planning your own care?

Comments

Yes

No

Not applicable/ don't know

1.1.3. Have you been given any advice/information about keeping yourself healthy in future?

Comments

Yes

No

Not applicable/ don't know

1.1.3.1. If yes, was this information given verbally or in written/pictorial form? (Please tick) VERBALLY WRITTEN PICTORIAL

1.1.3.2. If yes, was the information useful?

Comments

Yes

No

Not applicable/ don't know

1.2. The hospital/health service ensures that all types of services provided within the organisation are regularly monitored and evaluated.

1.2.1. Has anyone who works here asked you what you think about the services and care?

Comments

Yes
No
Not applicable/ don't know

1.2.1.1. If yes, did anyone tell you how they would use the information you gave?
Comments
Yes
No
Not applicable/ don't know

1.3. The hospital/health service has a Charter on Children's Rights, in line with the United Nations Convention on the Rights of the Child.
1.3.1. Have you been given a copy of a Charter on Children's Rights in hospital/health service?
Comments
Yes
No
Not applicable/ don't know

1.3.2. Have you seen anything in the hospital that explains your rights?
Comments
Yes
No
Not applicable/ don't know

1.3.3. Has any health worker talked to you about your rights?
Comments
Yes
No
Not applicable/ don't know

1.4. The hospital provides the possibility for parents/carers to stay with their child at all times during hospitalisation.
1.4.1. Did you want your parent to stay with you?
Comments
Yes
No
Not applicable/ don't know

1.4.2. If yes, did your parent stay with you all the time (including at night)?
Comments
Yes
No
Not applicable/ don't know

1.4.3. Did your parent stay with you during procedures (i.e. injections, blood extractions, etc)?
Comments
Yes
No
Not applicable/ don't know

1.4.4. If you had an operation, did your parent stay with you until you went to sleep (anaesthetised)?
Comments
Yes
No
Not applicable/ don't know

1.4.4.1. If no, would you have liked them to stay?
Comments
Yes
No
Not applicable/ don't know

1.4.5. Are you allowed to have your mobile phone or laptop with you?
Comments
Yes
No
Not applicable/ don't know

1.5. The hospital/health service pays special attention to the rights of adolescents to healthcare.
1.5.1. Did you use a medical service without a parent/carer?
Comments
Yes
No
Not applicable/ don't know

1.5.2. Can you get there by bus or train?
Comments
Yes
No
Not applicable/ don't know

1.5.3. Were the people you met friendly?
Comments
Yes
No

Not applicable/ don't know

1.5.4. Are you happy with the service that was provided to you?
Comments
Yes
No
Not applicable/ don't know

1.5.5. Do you feel that your privacy is respected in all aspects of care and treatment?
Comments
Yes
No
Not applicable/ don't know

1.5.6. Do you feel that your confidentiality is protected in all aspects of care and treatment?
Comments
Yes
No
Not applicable/ don't know
Please write here any other opinions and ideas you have about this right

Standard 2: Equality and non-discrimination
All children should be able to access healthcare and undergo any type of treatment without discrimination of any kind, irrespective of the child's or his or her parent's or legal guardian's race, colour, sex, language, religion, political or other opinion, national, ethnic or social origin, property, disability, birth or other status.

Sub Standards
2.1. The hospital/health service fulfils the rights of access of all children without discrimination of any kind.
2.1.1. Do you think that everyone in this hospital/health service is treated equally?
Comments
Yes
No
Not applicable/ don't know

2.2. The hospital/health service delivers a patient-centred care, which recognises not only the child's individuality and diverse circumstances and needs, but also those of his or her parents or carers.
2.2.1. Were you treated with respect?
Comments
Yes
No
Not applicable/ don't know

2.2.2. Do the health professionals always use your preferred name?

Comments
Yes
No
Not applicable/ don't know

2.2.3. If you needed, did the hospital/health service offer you translation services?
Comments
Yes
No
Not applicable/ don't know

2.3. The hospital/health service ensures the respect of children's privacy at all times.
2.3.1. Were you given the opportunity to be examined by a doctor of the same sex, upon your request?
Comments
Yes
No
Not applicable/ don't know

2.3.2. Were you given the opportunity to stay in a single or double room, upon your request?
Comments
Yes
No
Not applicable/ don't know

2.3.3. Were you given information in a private area?
Comments
Yes
No
Not applicable/ don't know

2.3.4. Were you examined in a private area?
Comments
Yes
No
Not applicable/ don't know

Please write here any other opinions and ideas you have about this right

Standard 3: Play and Learning
All children have opportunities for play, rest, leisure, recreation and their rights to education protected, suited to their age and condition, in spite of their healthcare needs.

Sub Standards

3.1. The hospital/health service ensures the right to play for all children without discrimination of any kind.
3.1.1. Have you been able to relax/play here?
Comments
Yes
No
Not applicable/ don't know

3.1.2. Was there a Playroom or separate space to play?
Comments
Yes
No
Not applicable/ don't know

3.1.2.1. Are there things to do here for a person of your age?
Comments
Yes
No
Not applicable/ don't know

3.1.3. Did anyone help you during play if you needed?
Comments
Yes
No
Not applicable/ don't know

3.1.4. Did the doctors or nurses use any type of play to help you during examination, treatment or procedures?
Comments
Yes
No
Not applicable/ don't know

3.2. The hospital/health service planning takes into account children's views of what is needed.
3.2.1. Has anyone who works here asked you what you think about the play here?
Comments
Yes
No
Not applicable/ don't know

3.3. The hospital/health service provides complementary play and educational activities.
3.3.1. Have you been able to continue your school work here?
Comments
Yes
No

Not applicable/ don't know

3.3.2. Has anyone told you about how to talk to a teacher here?
Comments
Yes
No
Not applicable/ don't know
Please write here any other opinions and ideas you have about this right

Standard 4: Information and Participation

All children receive information about their health problem, in ways that are understandable to them, can express their views and participate in decision-making about their care and treatment, in a manner consistent with their evolving capacities.

Sub Standards
4.1. The hospital/health service ensures an environment based on trust, information-sharing, the capacity to listen and sound guidance that is conducive to the child's effective participation.
4.1.1. Did you receive any information about your right to express your views and how this participation will take place?
Comments
Yes
No
Not applicable/ don't know

4.1.1.1. Have you been told that it is alright to ask the health professionals questions?
Comments
Yes
No
Not applicable/ don't know

4.1.1.2. Have you been told it is alright to tell the health professionals how you are feeling?
Comments
Yes
No
Not applicable/ don't know

4.1.1.3. Have your thoughts and opinions been asked for and listened to?
Comments
Yes
No
Not applicable/ don't know

4.1.2. Did all hospital/health service professionals introduce themselves to you and did they wear a name badge?
Comments

Yes
No
Not applicable/ don't know

4.1.3. Were you informed about your possibility to give informed consent to treatment?
Comments
Yes
No
Not applicable/ don't know

4.1.4. Did you give your informed consent for treatment (if you are old enough according to hospital/health service policy)?
Comments
Yes
No
Not applicable/ don't know

4.1.5. If you wanted to make a complaint about something here, would you know how to do that?
Comments
Yes
No
Not applicable/ don't know

4.2. The hospital/health service ensures that all appropriate staff has the skills to engage in dialogue and information-sharing with children of all ages and maturity.
4.2.1. Did you understand everything that you were told by the health professionals?
Comments
Yes
No
Not applicable/ don't know

4.2.2. Do you think that you were given enough information about what is wrong with you and what treatment you might need?
Comments
Yes
No
Not applicable/ don't know

4.3. The hospital/health service consults with children about their well being and about the development and improvement of health care services.
4.3.1. Have you ever participated in a consultation or other programme for evaluation and/or improving health care services?
Comments

Yes
No
Not applicable/ don’t know

4.3.1.1. If yes, did you receive clear feedback about how your contributions were used and/or influenced any outcomes?
Comments
Yes
No
Not applicable/ don’t know

4.3.1.2. If yes, did you feel your contributions influenced decisions?
Comments
Yes
No
Not applicable/ don’t know
Please write here any other opinions and ideas you have about this right

Standard 5: Safety and environment

All services for children are provided in an environment designed, furnished, staffed and equipped to meet their needs. Safety also includes aspects of cleanliness and food.
Sub Standards
5.1. The hospital/health service infrastructure is designed, furnished and equipped to meet children’s safety and mobility needs.
5.1.1. Do you feel safe here?
Comments
Yes
Partly
No

5.1.2. If you have mobility restrictions, have you been able to move around all areas of the building easily?
Comments
Yes
Partly
No
5.2. The hospital/health service policies and practice support the best possible nutrition for children.
5.2.1. Was free food provided to you during hospitalisation?
Comments
Yes
No
Not applicable/ don’t know

5.2.2. Did it come at the right times for you?
Comments
Yes
No
Not applicable/ don't know

5.2.3. Did you think the food was healthy?
Comments
Yes
No
Not applicable/ don't know

5.3. The hospital/health service policies and practice a clean environment for children at all times.
5.3.1. Do you think it is clean here?
Comments
Yes
No
Not applicable/ don't know

5.3.2. Did the health professionals always wash their hands before and after examining or treating you?
Comments
Yes
No
Not applicable/ don't know
Please write here any other opinions and ideas you have about this right

Standard 6: Pain management and palliative care
All children have the right to individualized, culturally and age appropriate prevention and management of pain and palliative care.

Sub Standards
6.1. The hospital/health service policy ensures the prevention and management of pain.
6.1.1. Were you in pain here?
Comments
Yes
No
Not applicable/ don't know

6.1.2. Were you given any treatment for this pain?
Comments
Yes
No
Not applicable/ don't know

6.1.3. Was anything else been done to make you feel more comfortable if you have pain (apart from medicine)?
Comments
Yes
No
Not applicable/ don't know
6.1.4. Did the health professionals ask you if you have pain?
Comments
Yes
No
Not applicable/ don't know
Please write here any other opinions and ideas you have about this right

Last question
What have been the good things about the services provided here?

What have been the bad things about the services provided being here?

If you were in charge, what are the first things you would change to make things better for children and adolescents?

THANK YOU
Printed name and initials of Interviewer___ Post held ___ Signature of Interviewer ___ Date ____
