# Supplementary material for: Perceptions and Expectations of Youth Regarding the Respect for Their Rights in the Hospital
Source: Children (Basel). 2024 Feb 9;11(2):222. doi: 10.3390/children11020222 (PMC10887615; doi:10.3390/children11020222)
Supplement: Supplementary file 1 [file children-11-00222-s001.zip › Table S1.pdf]

children

MDPI

**Table S1 .** Question 3 : Who did you play with?

| | **% µ (± SD)** |
|---|---|
| Parents/Relatives | 22,9 (± 15) |
| Teacher | 11,1 (± 21,97) |
| Volunteers | 15,5 (± 14,4) |
| Other Children | 45,4 (± 23) |
| Alone | 3.8 (± 4,8) |
| Doctors | 0.9 (± 1.5) |
