# Supplementary material for: Perceptions and Expectations of Youth Regarding the Respect for Their Rights in the Hospital
Source: Children (Basel). 2024 Feb 9;11(2):222. doi: 10.3390/children11020222 (PMC10887615; doi:10.3390/children11020222)
Supplement: Supplementary file 1 [file children-11-00222-s001.zip › Table S2.pdf]

children

MDPI

Table S2 Questionnaire 12-18 Years Standard 1: Quality Services For Children			
**STANDARD 1: QUALITY SERVICES FOR CHILDREN**	**% YES μ (± SD)**	**% NO μ (± SD)**	**% ? / N.A. μ (± SD)**
1.1.1. Do you think that you received the best care here?	92,00 (±4,62)	1,14 (±1,51)	6,86 (±4,86)
1.1.2. Do you feel you are included in planning your own care?	79,71 (±8,05)	11,71 (±5,24)	8,57 (±3,73)
1.1.3. Have you been given any advice/information about keeping yourself healthy in future?	79,43 (±9,09)	9,14 (±3,30)	11,43 (±4,86)
1.2. The hospital/health service ensures that all types of services provided within the organisation are regularly monitored and evaluated.			
1.2.1. Has anyone who works here asked you what you think about the services and care?	20,57 (±9,84)	76,86 (11,07)	2,57 (±2,30)
1.2.1.1. If *yes*, did anyone tell you how they would use the information you gave?	8,29 (±2,41)	83,42 (7,61)	8,29 (±5,62)
1.3. The hospital/health service has a Charter on Children's Rights, in line with the United Nations Convention on the Rights of the Child.			
1.3.1. Have you been given a copy of a Charter on Children's Rights in hospital/health service?	9,14 (±8,85)	85,14 (12,00)	5,72 (±4,91)
1.3.2. Have you seen anything in the hospital that explains your rights?	18,00 (±10,60)	78,86 (12,25)	3,14 (±2,15)
1.3.3. Has any health worker talked to you about your rights?	14,57 (±9,01)	83,14 (10,66)	2,29 (±1,86)
1.4. The hospital provides the possibility for parents/carers to stay with their child at all times during hospitalisation.			
1.4.1. Did you want your parent to stay with you?	96,29 (±4,49)	2,00 (2,65)	1,71 (±1,86)
1.4.2. If *yes*, did your parent stay with you all the time (including at night)?	96,86 (±4,16)	3,14 (4,16)	0,00 (±0,00)
1.4.3. Did your parent stay with you during procedures (i.e. injections, blood extractions, etc)?	95,43 (±3,95)	4,00 (4,04)	0,57 (±0,76)
1.4.4. If you had an operation, did your parent stay with you until you went to sleep (anaesthetised)?	56,57 (±20,12)	16,29 (10,65)	27,14 (±17,63)
1.4.4.1. If *no*, would you have liked them to stay?	77,71 (±17,77)	4,57 (3,73)	17,72 (±18,22)
1.4.5. Are you allowed to have your mobile phone or laptop with you?	96,00 (±4,47)	2,00 (2,65)	2,00 (±1,91)

1.5. The hospital/health service pays special attention to the rights of adolescents to healthcare.			
1.5.1. Did you use a medical service without a parent/carer?	7,71 (±3,67)	89,43 (5,19)	2,86 (±1,90)
1.5.2. Can you get there by bus or train?	58,00 (±15,37)	8,00 (1,91)	34,00 (±15,73)
1.5.3. Were the people you met friendly?	94,00 (±3,79)	2,29 (2,27)	3,71 (±1,91)
1.5.4. Are you happy with the service that was provided to you?	90,29 (±6,62)	2,29 (1,57)	7,42 (±3,78)
1.5.5. Do you feel that your privacy is respected in all aspects of care and treatment?	89,71 (±5,73)	4,00 (2,24)	6,29 (±3,70)
1.5.6. Do you feel that your confidentiality is protected in all aspects of care and treatment?	87,71 (±7,80)	3,43 (±2,36)	8,86 (±6,02)
TOTAL RIGHTS	**63,40 (±8,05)**	**28,54 (±5,35)**	**8,06 (±5,19)**
