# Supplementary material for: Perceptions and Expectations of Youth Regarding the Respect for Their Rights in the Hospital
Source: Children (Basel). 2024 Feb 9;11(2):222. doi: 10.3390/children11020222 (PMC10887615; doi:10.3390/children11020222)
Supplement: Supplementary file 1 [file children-11-00222-s001.zip › Table S3.pdf]

children MDPI

**Table S3** Questionnaire 12-18 Years Standard 2 : Equality And Non-Discrimination			
**STANDARD 2: EQUALITY AND NON-DISCRIMINATION**	**% YES μ (± SD)**	**% NO μ (± SD)**	**% ? / N.A. μ (± SD)**
2.1. The hospital/health service fulfils the rights of access of all children without discrimination of any kind.			
2.1.1. Do you think that everyone in this hospital/health service is treated equally?	84,86 (±8,34)	6,57 (±4,99)	8,57 (±5,74)
2.2. The hospital/health service delivers a patient-centred care, which recognises not only the child's individuality and diverse circumstances and needs, but also those of his or her parents or carers.			
2.2.1. Were you treated with respect?	97,43 (±1,89)	0,00 (±0,00)	2,57 (±1,89)
2.2.2. Do the health professionals always use your preferred name?	76,57 (±9,20)	14,00 (±7,64)	9,43 (±5,36)
2.2.3. If you needed, did the hospital/health service offer you translation services?	4,86 (±2,79)	13,43 (±6,45)	81,71 (±7,97)
2.3. The hospital/health service ensures the respect of children's privacy at all times.			
2.3.1. Were you given the opportunity to be examined by a doctor of the same sex, upon your request?	24,29 (±11,61)	31,14 (±13,73)	44,57 (±12,89)
2.3.2. Were you given the opportunity to stay in a single or double room, upon your request?	12,00 (±7,77)	39,43 (±14,44)	48,57 (±13,15)
2.3.3. Were you given information in a private area?	67,14 (±14,07)	21,71 (±12,69)	11,15 (±3,99)
2.3.4. Were you examined in a private area?	78,57 (±7,67)	14,57 (±6,65)	6,86 (±2,51)
TOTAL RIGHTS	**55,71 (±7,92)**	**17,61 (±8,32)**	**26,68 (±6,69)**
