# Supplementary material for: Perceptions and Expectations of Youth Regarding the Respect for Their Rights in the Hospital
Source: Children (Basel). 2024 Feb 9;11(2):222. doi: 10.3390/children11020222 (PMC10887615; doi:10.3390/children11020222)
Supplement: Supplementary file 1 [file children-11-00222-s001.zip › Table S4.pdf]

children MDPI

**Table S4** Questionnaire 12-18 Years Standard 3 : Play And Learning			
**STANDARD 3: PLAY AND LEARNING**	**% YES μ (± SD)**	**% NO μ (± SD)**	**% ? / N.A. μ (± SD)**
3.1. The hospital/health service ensures the right to play for all children without discrimination of any kind.			
3.1.1. Have you been able to relax/play here?	78,57 (±7,97)	18,29 (±8,91)	3,14 (±2,30)
3.1.2. Was there a Playroom or separate space to play?	75,14 (±16,75)	7,14 (±3,82)	17,72 (±13,72)
3.1.2.1. Are there things to do here for a person of your age?			
3.1.3. Did anyone help you during play if you needed?	40,29 (±14,75)	14,00 (±3,11)	45,71 (±12,17)
3.1.4. Did the doctors or nurses use any type of play to help you during examination, treatment or procedures?	38,57 (±15,81)	38,29 (±13,66)	23,14 (±12,51)
3.2. The hospital/health service planning takes into account children's views of what is needed.			
3.2.1. Has anyone who works here asked you what you think about the play here?	11,14 (±7,61)	81,43 (±7,80)	6,29 (±3,29)
3.3. The hospital/health service provides complementary play and educational activities.			
3.3.1. Have you been able to continue your school work here?	35,71 (±13,04)	57,71 (±14,99)	6,57 (±3,61)
3.3.2. Has anyone told you about how to talk to a teacher here?	28,86 (±12,70)	60,00 (±16,22)	11,14 (±3,85)
TOTAL RIGHTS	**44,04 (±12,66)**	**39,55 (±9,79)**	**16,41 (±7,35)**
