# Supplementary material for: Perceptions and Expectations of Youth Regarding the Respect for Their Rights in the Hospital
Source: Children (Basel). 2024 Feb 9;11(2):222. doi: 10.3390/children11020222 (PMC10887615; doi:10.3390/children11020222)
Supplement: Supplementary file 1 [file children-11-00222-s001.zip › Table S5.pdf]

children MDPI

**Table S5** Questionnaire 12-18 Years Standard 4: Information And Participation			
**STANDARD 4: INFORMATION AND PARTICIPATION**	**% YES µ (± SD)**	**% NO µ (± SD)**	**% ? / N.A. µ (± SD)**
4.1. The hospital/health service ensures an environment based on trust, information-sharing, the capacity to listen and sound guidance that is conducive to the child's effective participation.			
4.1.1. Did you receive any information about your right to express your views and how this participation will take place?	29,14 (±11,94)	63,43 (±11,48)	7,43 (±3,55)
4.1.1.1. Have you been told that it is alright to ask the health professionals questions?	74,00 (±7,87)	19,71 (±7,47)	6,29 (±3,82)
4.1.1.2. Have you been told it is alright to tell the health professionals how you are feeling?	96,00 (±4,47)	4,00 (±4,47)	0,00 (±0,00)
4.1.1.3. Have your thoughts and opinions been asked for and listened to?	86,57 (±6,32)	5,71 (±3,39)	7,71 (±2,38)
4.1.2. Did all hospital/health service professionals introduce themselves to you and did they wear a name badge?	91,71 (±5,24)	4,86 (±4,31)	3,43 (±3,10)
4.1.3. Were you informed about your possibility to give informed consent to treatment?	62,86 (±13,50)	25,43 (±12,98)	11,71 (±3,02)
4.1.4. Did you give your informed consent for treatment *(if you are old enough according to hospital/health service policy)*?	63,43 (±10,24)	25,71 (±12,10)	10,86 (±7,72)
4.1.5. If you wanted to make a complaint about something here, would you know how to do that?	56,57 (±6,34)	34,86 (±6,92)	8,57 (±5,67)
4.2. The hospital/health service ensures that all appropriate staff has the skills to engage in dialogue and information-sharing with children of all ages and maturity.			
4.2.1. Did you understand everything that you were told by the health professionals?	64,57 (±15,76)	30,57 (±13,74)	4,86 (±3,26)
4.2.2. Do you think that you were given enough information about what is wrong with you and what treatment you might need?	77,43 (±9,11)	18,00 (±10,82)	4,57 (±4,82)
4.3. The hospital/health service consults with children about their well being and about the development and improvement of health care services.			
4.3.1. Have you ever participated in a consultation or other programme for evaluation and/or improving health care services?	16,86 (±8,16)	79,14 (±8,18)	4,00 (±2,65)
4.3.1.1. If *yes*, did you receive clear feedback about how your contributions were used and/or influenced any outcomes?	9,14 (±6,90)	62,29 (±16,78)	28,57 (±16,94)
4.3.1.2. If *yes*, did you feel your contributions influenced decisions?	17,43 (±9,98)	49,43 (±13,49)	33,14 (±16,69)
TOTAL RIGHTS	**57,36 (±8,91)**	**32,55 (±9,70)**	**10,09 (±5,66)**
