# Supplementary material for: Perceptions and Expectations of Youth Regarding the Respect for Their Rights in the Hospital
Source: Children (Basel). 2024 Feb 9;11(2):222. doi: 10.3390/children11020222 (PMC10887615; doi:10.3390/children11020222)
Supplement: Supplementary file 1 [file children-11-00222-s001.zip › Table S6.pdf]

children

MDPI

**Table S6** Questionnaire 12-18 Years Standard 5: Safety And Environment			
**STANDARD 5: SAFETY AND ENVIRONMENT**	**% YES μ (± SD)**	**% NO μ (± SD)**	**% ? / N.A. μ (± SD)**
5.1. The hospital/health service infrastructure is designed, furnished and equipped to meet children's safety and mobility needs.			
5.1.1. Do you feel safe here?	91,71 (±5,30)	4,86 (±4,16)	3,43 (±4,54)
5.1.2. If you have mobility restrictions, have you been able to move around all areas of the building easily?	71,71 (±12,81)	7,14 (±4,31)	21,14 (±10,61)
5.2. The hospital/health service policies and practice support the best possible nutrition for children.			
5.2.1. Was free food provided to you during hospitalisation?	97,71 (±3,02)	2,29 (±3,02)	0,00 (±0,00)
5.2.2. Did it come at the right times for you?	58,57 (±11,40)	37,71 (±10,96)	3,71 (±2,04)
5.2.3. Did you think the food was healthy?	85,43 (±6,24)	8,29 (±4,02)	6,29 (±4,88)
5.3. The hospital/health service policies and practice a clean environment for children at all times.			
5.3.1. Do you think it is clean here?	75,71 (±9,14)	16,86 (±8,72)	7,43 (±3,73)
5.3.2. Did the health professionals always wash their hands before and after examining or treating you?	51,14 (±12,86)	6,57 (±4,27)	42,29 (±13,82)
TOTAL RIGHTS	**76,00 (±8,68)**	**11,96 (±5,64)**	**12,04 (±5,66)**
