# Supplementary material for: Perceptions and Expectations of Youth Regarding the Respect for Their Rights in the Hospital
Source: Children (Basel). 2024 Feb 9;11(2):222. doi: 10.3390/children11020222 (PMC10887615; doi:10.3390/children11020222)
Supplement: Supplementary file 1 [file children-11-00222-s001.zip › Table S7.pdf]

Table S7 Questionnaire 12-18 Years Standard 6: Pain Management And Palliative Care

**STANDARD 6: PAIN MANAGEMENT AND PALLIATIVE CARE**	**% YES μ (± SD)**	**% NO μ (± SD)**	**% ? / N.A. μ (± SD)**
6.1. The hospital/health service policy ensures the prevention and management of pain.			
6.1.1. Were you in pain here?	65,43 (±11,63)	31,71 (±11,82)	2,86 (±1,25)
6.1.2. Were you given any treatment for this pain?	78,29 (±9,46)	10,57 (±3,25)	11,14 (±7,04)
6.1.3. Was anything else been done to make you feel more comfortable if you have pain (apart from medicine)?	68,86 (±12,29)	18,00 (6,81)	13,14 (7,91)
6.1.4. Did the health professionals ask you if you have pain?	94,57 (±5,50)	5,43 (±5,50)	0,00 (±0,00)
TOTAL RIGHTS	**76,79 (±9,89)**	**16,43 (±6,85)**	**6,79 (±4,05)**
