# Supplementary material for: Perceptions and Expectations of Youth Regarding the Respect for Their Rights in the Hospital
Source: Children (Basel). 2024 Feb 9;11(2):222. doi: 10.3390/children11020222 (PMC10887615; doi:10.3390/children11020222)
Supplement: Supplementary file 1 [file children-11-00222-s001.zip › Table S8.pdf]

**TABLE S8** Types of feeling in 144 children’s drawings		
**FEELING**	**POSITIVE FEELINGS (N)**	**NEGATIVE FEELINGS (N)**
	Joy / Confidence (23)	Mood fluctuations (17)
	Welcome / Hospitality (18)	Uncertainty/Anxiety (17)
	Joy/ Liveliness (15)	Sadness (14)
	Optimism (9)	Need for protection (9)
	Stability/Safety (8)	Closed environment refusal (7)
	Joy/Fun (7)	
**N TOTAL**	80	64
**%**	55%	45%
